# Enhanced electrochemical sensing of methyl parathion using AgNPs@IL/GO nanocomposites in aqueous matrices†

**DOI:** 10.1039/d4na00919c

**Published:** 2025-02-24

**Authors:** Saddam Weheabby, Ziyuan Liu, Igor A. Pašti, Vladimir Rajić, Marcio Vidotti, Olfa Kanoun

**Affiliations:** a Chemnitz University of Technology, Measurement and Sensor Technology Chemnitz 09126 Germany saddam.weheabby@etit.tu-chemnitz.de; b University of Belgrade – Faculty of Physical Chemistry Studentski Trg 12-16 Belgrade 11158 Serbia; c University of Belgrade, VINČA Institute of Nuclear Sciences – National Institute of the Republic of Serbia Mike Petrovica Alasa 12-14 11000 Belgrade Serbia; d Grupo de Pesquisa em Macromoléculas e Interfaces, Universidade Federal do Paraná (UFPR) CxP 19032 Curitiba 81531-980 PR Brazil

## Abstract

Methyl parathion (MP) is a widely used pesticide; it is recognized as being toxic to both target and non-target species, posing serious risks to environmental and human health. Monitoring and controlling MP residues is thus essential, necessitating the development of innovative sensors that are highly sensitive, selective, and reproducible. In the present study, an efficient electrochemical MP sensor is proposed based on silver nanoparticles (AgNPs) in conjunction with graphene oxide/ionic liquid (GO/IL) on screen printed electrodes (AgNPs@GO/IL@SPCE). The AgNPs were synthesized *via* a cost-effective wet-chemical process and characterized using UV-Vis spectroscopy and transmission electron microscopy (TEM). The modified electrodes were characterized by scanning electron microscopy (SEM) and energy-dispersive X-ray spectroscopy (EDX). The active surface area and charge transfer were examined by cyclic voltammetry (CV) and electrochemical impedance spectroscopy (EIS), respectively. The modified electrodes' electrocatalytic performance towards the reduction of MP was investigated by CV, complemented by semiempirical quantum chemistry calculations to elucidate the interaction and the electrochemical reduction mechanism of MP. The sensor demonstrates a remarkable limit of detection of 0.009 μmol L^−1^ within a linear range of 0.025 to 200 μmol L^−1^. It has an excellent analytical performance in terms of selectivity, reproducibility, and long-term stability over 60 days. The designed sensor was effectively used to inspect MP in groundwater and surface water samples, with recovery values ranging from 95.60% to 99.68%.

## Abstract 2

A high-performance electrochemical sensor for methyl parathion (MP) detection was developed using silver nanoparticles (AgNPs) integrated with graphene oxide/ionic liquid (GO/IL) on a screen-printed electrode (AgNPs@GO/IL@SPCE) for enhanced sensitivity and stability.

## Graphical abstract

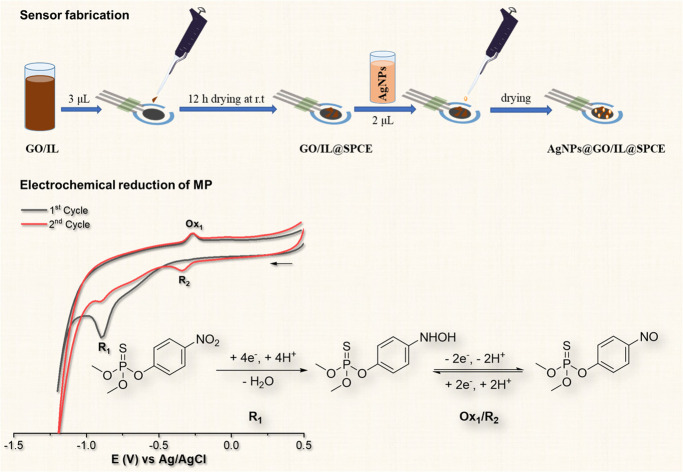

## 1. Introduction

Organophosphates are a broad class of chemical compounds that can be easily produced by the esterification reaction of phosphoric acid and alcohol. Owing to their chemical attributes, they have been utilized in various applications such as flame retardants, fuel additives, lubricants, plasticizers, pharmaceuticals, and pesticides.^1^ Organophosphate pesticides (OPs) account for approximately 40% of the total pesticides used in agricultural production worldwide.^2^ They are highly toxic and have harmful effects on the environment, animals, and human health. Frequent exposure to low doses of OPs can lead to neurological disorders, including dementia, autism, cognitive development problems, Parkinson's disease, and neuropsychiatric disorder.^3^ Methyl parathion (MP) is an important OP commonly used worldwide to control insects in crops due to its high efficacy, ease of degradation, and low cost.^4^ However, MP entering the human body leads to several health problems.^5^ Therefore, several investigations have been addressing ways to determine and control its concentration in soil, food, and water matrices.^6–8^ Analytical methods, including gas chromatography-mass spectrometry (GC-MS), solid-phase extraction, and high-performance liquid chromatography (HPLC), are the gold standard techniques for the determination of MP.^9–11^ Despite their high accuracy, precision, and sensitivity, these technologies are costly, necessitating specialized staff and a lot of time and effort, as well as specific conditions and environments.^12^ Thus, it is worth investigating novel ways of designing novel MP sensors suitable for on-site applications and overcoming these obstacles. Among several sensor principles, electrochemical sensors using appropriate sensing materials have a high potential to meet these requirements due to their high sensitivity, low limit of detection and cost effectiveness.^13–15^

Numerous sensing materials have been proposed, ranging from nanomaterials to (macro)molecules capable of reducing and detecting MP. Examples include Ag@graphene,^16^ CuNPs@GR-MIP,^17^ Au–Ag@BSA,^18^ [Co(bpy)_3_]@rGO,^19^ pillar[5]arene@AuNPs@rGO.^20^ Carbon-based nanomaterials, including graphene, carbon nanotubes (CNT), and graphene oxide (GO), have gained great attention in electrochemical sensors due to their large surface area, electronic structure, and electrochemical performance.^21–24^ Their physicochemical properties can be even enhanced once combined with other materials. For instance, the high oxygen content of GO makes it hydrophilic and suitable for forming a stable aqueous suspension. Nonetheless, to improve its long-term stability, it is possible to take advantage of the large delocalized π-electron system of GO by forming a strong π–π interaction with other materials, such as ionic liquids.^25,26^ On the other side, the grafting of the GO surface by metallic nanoparticles greatly improves its catalytic activity.^27^ Noble metal nanoparticles are highly effective catalysts, offering a high surface-to-volume ratio and strong plasmonic behavior. Among these, silver nanoparticles (AgNPs) are often the preferred option due to their cost-effectiveness, making them an economical choice for many applications.^28^

In light of what has been mentioned, in the present study, we aim at realizing and characterizing an MP sensor for aqueous media based on a screen-printed electrode (SPCE) modified with a highly stable composite of GO and ionic liquid (GO/IL) and silver nanoparticles (AgNPs). AgNPs are synthesized using a simple, cost-effective wet-chemical process, ensuring straightforward, economical, and environmentally friendly sensor. To elucidate how MP interacts with sensor materials, computational chemistry is used to investigate the adsorption of MP on the sensor surface. The investigation addresses also real sample analysis to quantify the MP in real groundwater and surface water.

## 2. Experimental

### 2.1. Chemicals and reagents

Methyl parathion, glyphosate, chlorpyrifos, pirimicarb, and malathion were purchased from Sigma-Aldrich, Germany. Graphene oxide (GO) was purchased from Graphene Supermarket, USA. 1-Butyl-3-methylimidazolium hexafluorophosphate (IL), silver nitrate (AgNO_3_), anhydrous trisodium citrate (Na_3_C_6_H_5_O_7_), potassium ferrocyanide (K_4_[Fe(CN)_6_]), potassium ferricyanide (K_3_[Fe(CN)_6_]), sodium phosphate monobasic monohydrate (NaH_2_PO_4_·H_2_O), sodium phosphate dibasic heptahydrate (Na_2_HPO_4_·7H_2_O), sodium sulfate (NaSO_4_), sodium carbonate (Na_2_CO_3_), potassium chloride (KCl), sodium chloride (NaCl), and sodium nitrite (NaNO_2_) were purchased from Sigma-Aldrich. All the chemicals used in this work were of analytical grade and used as received.

### 2.2. Synthesis of silver nanoparticles

The synthesis of silver nanoparticles involved the use of silver nitrate (AgNO_3_) as a precursor and trisodium citrate (Na_3_C_6_H_5_O_7_) as a reducing agent in a molar ratio of 1 : 10.^29^ Briefly, an aqueous solution of AgNO_3_ (25 mL) was heated to 70 °C and added dropwise to a hot aqueous solution (70 °C) of Na_3_C_6_H_5_O_7_ (25 mL) while continuously stirring. The reaction mixture began to change from colorless to a pale yellow, indicating the formation of silver nanoparticles. At this point, the heating was discontinued, but the stirring was continued until the reaction mixture reached room temperature.

### 2.3. Instrumentation

Ultraviolet-visible (UV-Vis) spectra of the AgNP solution were recorded at room temperature using an Agilent Cary 60 UV-Vis spectrophotometer. Transmission electron microscopy analyses of AgNPs were performed using a JEOL JEM 1200EX-II transmission electron microscope. For sample preparation, the samples were dispersed in ultrapure water (diluted 10×) under ultrasonic bath treatment for 10 minutes, then an aliquot of 5 μL was dropped onto the TEM grid and immediately frozen with liquid nitrogen. The solvent was sublimated under low pressure and room temperature to avoid any aggregation of the particles. Each sample underwent freeze-drying at −52 °C under a vacuum of 190 mmHg for 24 hours, and at least three different samples were analyzed to ensure homogeneity and reproducibility of the synthesis. Fourier transform infrared spectroscopy study (FT-IR) was done using an Avatar 370 spectrometer from Thermo Nicolet. Raman analysis of the modified electrodes was done using a DXR Raman microscope (Thermo Scientific). Spectra were excited using a 532 nm laser, with a power of 2.0 mW. Scanning Electron Microscopy (SEM) and Energy Dispersive X-ray analysis (EDX) were done to assess the morphology and the elemental content of sensor electrodes by a Phenom ProX electron microscope (Phenom, the Netherlands). The EDX analysis was achieved without depositing the conductive layer on the electrodes. The EDX mapping was done at a magnification of ×2500 to properly average the elemental content of the electrodes' surface layers. For SEM analysis, the electrodes were coated with a thin Cu layer. An acceleration voltage of 15 keV was used. Additional SEM characterization of the sensor electrodes was done using a Scios 2 DualBeam FIB-SEM (Thermo Fisher Scientific). Before the analysis, the samples were covered with a thin Au layer. EDX characterization was repeated at least three times on different spots to obtain a realistic elemental composition of the electrodes. Electrochemical analyses, including cyclic voltammetry (CV), square wave voltammetry (SWV), and electrochemical impedance spectroscopy (EIS), were conducted utilizing the PalmSens4 portable potentiostat (Palmsens BV, GA Houten, Netherlands). Screen-printed carbon electrodes (SPCE, geometric area of the working electrode: 7 mm^2^) were used as the platform for electrochemical tests and sensor characterization, which consists of a carbon counter electrode, graphite working electrode, and an Ag/AgCl reference electrode.

### 2.4. Theoretical calculations

Semiempirical calculations were done using MOPAC2016 code^30^ with the PM7 method,^31^ and they were managed using MoCalc2012 code.^32^ The full structural relaxation was done. Visualization was done using Jmol^33^ and VESTA.^34^ We have analyzed the interactions of ML with the Ag@GO/IL system. A GO sheet was used with one island of clustered OH groups and a part of the preserved sp^2^ domain. One formula unit of IL was added to the system to analyze its effects on the ML interaction with the Ag cluster. Calculations were done with the implicit addition of water as a solvent using the COSMO model.^35^

### 2.5. Preparation of the modified electrode

**Fig. 1:**
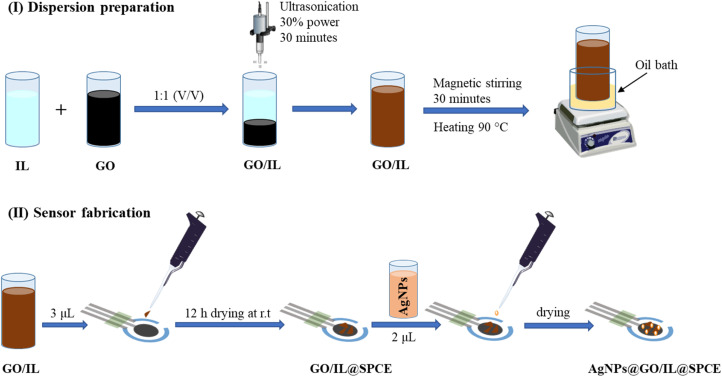
Dispersion preparation (I) and sensor fabrication (II).

The GO/IL composite was prepared in analogy to a published procedure.^36^ In a typical experiment, an aqueous suspension of GO and 1-butyl-3-methylimidazolium hexafluorophosphate (IL) was mixed using (5 mL, 0.5 mg mL^−1^) and (5 mL, 2 mol L^−1^), respectively (Fig. 1).

To ensure uniformity and homogeneity, the resulting mixture was sonicated at 30% of maximum power for 30 minutes using an ultrasonic sonicator (GM 3200, Bandelin Electronic, Berlin, Germany). After that, it was subjected to magnetic stirring and heating in an oil bath at 90 °C for 1 hour to ensure better homogeneity and enhance the long-term stability of the composite. Subsequently, 3 μL of GO/IL was applied on the working electrode of the SPCE and allowed to dry at ambient temperature for 12 h, forming the GO/IL@SPCE electrode. Subsequently, 2 μL of AgNPs were added to the modified GO/IL@SPCE electrode, resulting in the formation of the desired electrode, which we will henceforth refer to as AgNPs@GO/IL@SPCE. The ratio of the AgNPs to the GO/IL composite has been investigated and optimized by controlling the oxidation current of the [Fe(CN)_6_]^3−/4−^ system to ensure the best possible electrocatalytic behavior of the modifier materials (Fig. S1†). The ratio was found to be 2 : 3 for AgNPs to GO/IL, and this ratio was consistently applied throughout the study.

### 2.6. Real sample analysis

The applicability of the AgNPs@GO/IL@SPCE sensor was examined by detecting MP in samples of groundwater and surface water. Groundwater samples were provided by the Saxony State Office for Environment, Agriculture, and Geology in Saxony, Germany, while surface water samples were collected from the Chemnitz river and filtered through Whatman filter paper. A 20 mL aliquot of the real sample was spiked with a known concentration of MP (500 μmol L^−1^). The pH was optimized to 7 to align with the optimal conditions for electrochemical activity. The sample was then diluted to the desired concentrations (5, 20, and 40 μmol L^−1^). A volume of 100 μL of the prepared solutions was subsequently deposited onto the modified SPCE for recovery analysis.

## 3. Results and discussion

### 3.1. Characterization of AgNPs

The UV-Vis analysis of the prepared silver nanoparticle (AgNP) suspension exhibits an intense absorption peak at 430 nm, confirming the successful formation of AgNPs.

**Fig. 2:**
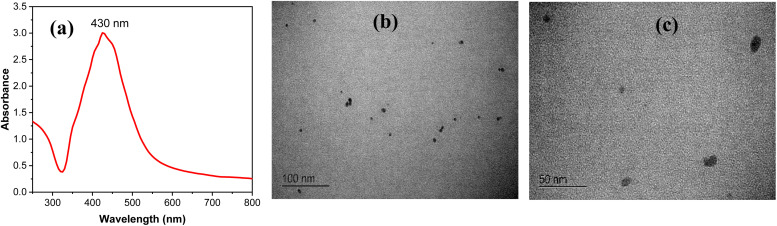
(a) UV-Vis spectrum of AgNPs. (b) and (c) Representative images from TEM analysis of AgNPs at different magnifications; more images are provided in the ESI (Fig. S2†).

This characteristic band is indicative of the surface plasmon resonance of the nanoparticles,^37^ as shown in Fig. 2a. Additionally, TEM was employed to conduct an in-depth analysis of the surface morphology of the synthesized AgNPs. The TEM images reveal that the AgNPs possess a spherical-like shape with a uniform and well-dispersed appearance (Fig. 2b and c). The average particle size was found to be around 20 nm.

### 3.2. FT-IR and Raman analysis of the modified electrode

**Fig. 3:**
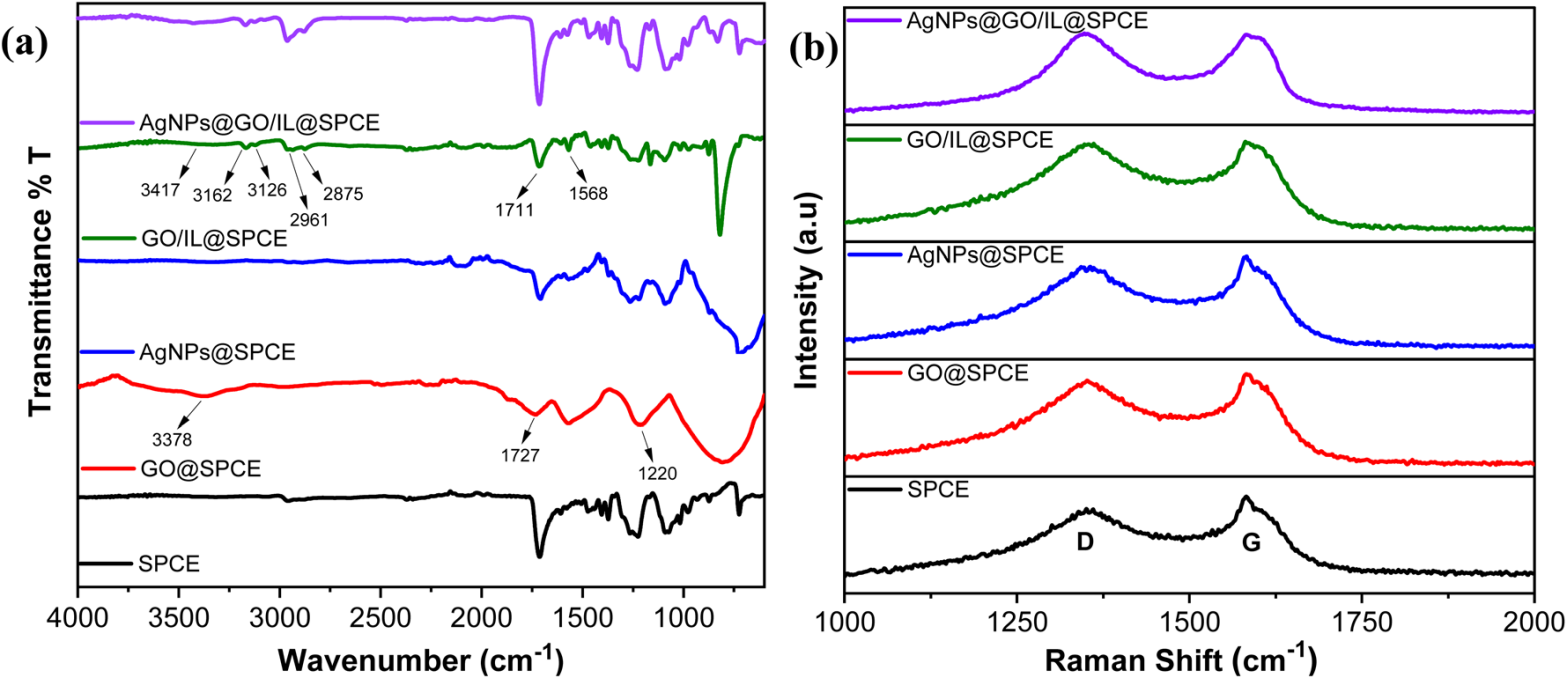
(a) FT-IR; (b) Raman spectra of SPCE, GO@SPCE, AgNPs@SPCE, GO/IL@SPCE, and AgNPs@GO/IL@SPCE.

The successful formation of the nanocomposite and the interactions of modifier materials have been confirmed by IR (Fig. 3a) and Raman analysis (Fig. 3b). The FTIR spectrum of the SPCE shows characteristic absorption peaks in the range of 1000–1600 cm^−1^, which basically originate from the substrate materials used in the fabrication of SPCE as presented in our previous study.^37^ However, the modification of SPCE with GO resulted in changes in the IR spectrum, characterized by the stretching vibrations of the epoxy group (C–O, 1220 cm^−1^), carbonyl group (C=O, 1727 cm^−1^), and hydroxyl groups (O–H, 3375 cm^−1^). These changes confirm the presence of oxygen-containing functional groups and thus successful integration of GO.^36^ The integration of AgNPs led to a slight change in the spectrum compared to SPCE and introduced broad peaks in the 1200–1500 cm^−1^ region, confirming the integration of AgNPs on SPCE.

In GO/IL@SPCE, the combination of the ionic liquid with GO resulted in an IR spectrum showing peaks corresponding to those on the GO surface, with slight shifts observed. Additionally, peaks at 1568 cm^−1^, 2963 and 2874 cm^−1^, as well as 3162 and 3126 cm^−1^ are attributed to the C=C, symmetric/asymmetric CH_2_, and symmetric/asymmetric CH stretching vibrations of the imidazolium ring, respectively.^36,38^ These changes suggest the successful integration of GO and ionic liquid through π–π interactions.^39^ Furthermore, the IR spectrum of AgNPs@GO/IL@SPCE shows a representative change in peak intensity compared to GO/IL@SPCE, suggesting the successful deposition of AgNPs.

Raman analysis provides valuable insights into structural alterations and defects in the materials during the electrode modification process. Fig. 3b presents the Raman spectra for SPCE, GO@SPCE, AgNPs@SPCE, GO/IL@SPCE, and AgNPs@GO/IL@SPCE. The two main peaks, the D band (associated with defects or disorder) and the G band (indicative of graphitic sp^2^ bonding), along with the *I*_D_/*I*_G_ ratio, are clearly identified and summarized in Table 1. The deposition of GO onto the SPCE surface introduced additional defects, as reflected by the increased *I*_D_/*I*_G_ value compared to that of the bare SPCE. The AgNPs@SPCE electrode shows a slight increase in the *I*_D_/*I*_G_ ratio, suggesting minor structural disorder while demonstrating the successful integration of AgNPs onto the SPCE surface. The *I*_D_/*I*_G_ ratio of GO/IL@SPCE is higher than that of GO@SPCE, highlighting additional disorder introduced during modification, likely due to π–π interactions between the ionic liquid and the graphene oxide surface.^40^ The Raman spectrum of AgNPs@GO/IL@SPCE reveals further increases in defects and disorder, as evidenced by shifts in the D and G bands and an enhanced *I*_D_/*I*_G_ ratio. This can be attributed to the interactions between AgNPs and the GO/IL composite (Fig. 3b, Table 1). The FT-IR and Raman analyses are consistent, confirming the successful integration and interaction of the modifier electrode materials during the modification process. This enhances the surface chemistry and available interaction sites, ultimately improving the electrochemical behavior.

**Table 1:** Summary of Raman spectra

Sensor electrode	D band (cm^−1^)	G band (cm^−1^)	*I*_D_/*I*_G_ (*n* = 3)
SPCE	1352	1581	0.83
GO@SPCE	1350	1582	0.91
AgNPs@SPCE	1354	1582	0.88
GO/IL@SPCE	1355	1582	0.98
AgNPs@GO/IL@SPCE	1347	1580	1.03

### 3.3. SEM and EDX analysis of the modified electrode

SEM images (Fig. S3†) show that the overall morphologies of the starting SPCE electrodes were preserved in each modification step. For the GO@SPCE electrode (Fig. S3a†), there is a thin layer of GO covering the surface, which is visible only after depositing the Cu layer on the electrode (plasma coating) as the GO layer is transparent for electrons due to the thickness. When GO/IL@SPCE is inspected (Fig. S3b†), small coils have been observed, which are crystallized/agglomerated IL.

**Fig. 4:**
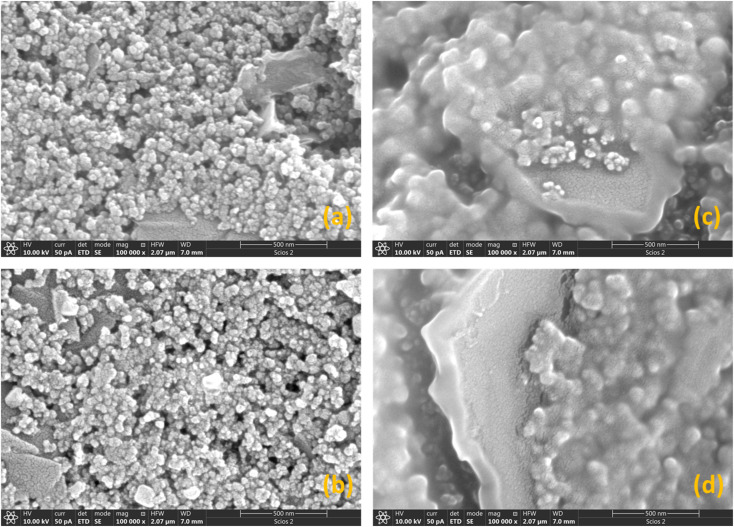
High-resolution images (magnification × 100 000) of (a) GO@SPCE, (b) AgNPs@SPCE, (c) GO/IL@SPCE, and (d) AgNPs@GO/IL@SPCE sensor electrodes (recorded after covering the electrodes with a thin Au layer).

**Fig. 5:**
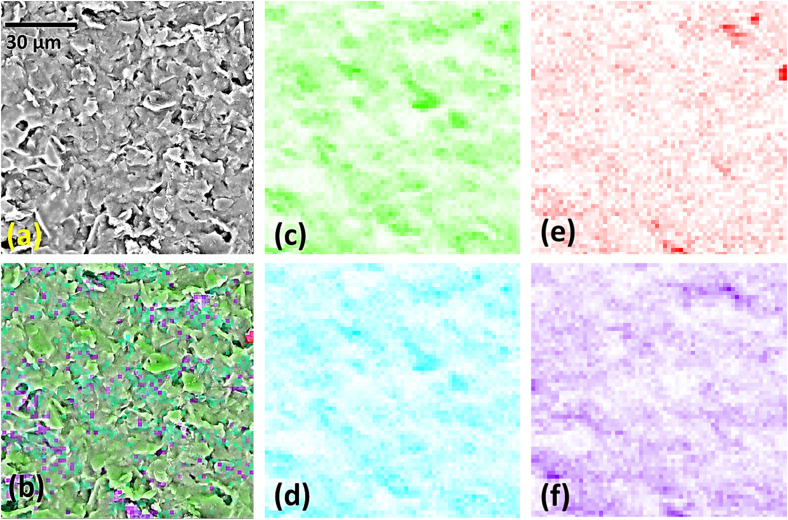
AgNPs@GO/IL@SPCE electrode under the magnification of ×2500 (a), full EDX map (b), and maps of selected elements: (c) carbon, (d) silver, (e) oxygen, and (f) fluorine. The analysis was done without Cu coating. The map resolution was 64 × 64, limited by a relatively low conductivity of the electrodes without conductive coating, causing significant electron accumulation during the analysis.

This surface modification is also only visible for Cu-coated electrodes due to the electron transparency. For AgNPs@SPCE and the AgNPs@GO/IL@SPCE, we have observed sparsely distributed AgNPs (small bright spots in Fig. S3c and d†). This finding suggests that the concentration of Ag is very low, which was confirmed by EDX analysis (Table 2). High-resolution SEM images are presented in Fig. 4. The EDX mapping of the AgNPs@GO/IL@SPCE electrode (Fig. 5) suggests there are some agglomerated IL, which is seen from some highly concentrated fluorine regions, and the same variations in elemental distributions were observed for carbon and oxygen.

**Table 2:** Elemental content of studied sensor electrodes

Sensor electrode	C (wt%)	O (wt%)	Ag (wt%)	F (wt%)	P (wt%)
SPCE	89.2 ± 0.7	5.5 ± 0.3	—	—	—
GO@SPCE	83.6 ± 0.6	13.1 ± 0.5	—	—	—
AgNPs@SPCE	80.5 ± 0.6	13.0 ± 0.5	0.1 ± 0.1	—	—
GO/IL@SPCE	74.2 ± 0.5	5.4 ± 0.3	—	12.5 ± 0.4	2.6 ± 0.5
AgNPs@GO/IL@SPCE	65.8 ± 0.6	4.7 ± 0.4	0.1 ± 0.1	14.9 ± 0.3	2.7 ± 0.6

Table 2 outlines the atomic composition of the studied modified SPCEs. In the case of GO/IL@SPCE, successful functionalization is confirmed by a large fluorine content, while phosphorus is also observed in the cases of IL-containing electrodes. However, both the AgNP@SPCE and the AgNPs@GO/IL@SPCE sensor display very low Ag content, which closely agrees with the SEM results. In both cases, the Ag content is only 0.1 wt%. This suggests that very small amounts of silver are sufficient to boost the sensing performance of the modified electrodes appreciably.

### 3.4. Electrochemical characterization

**Fig. 6:**
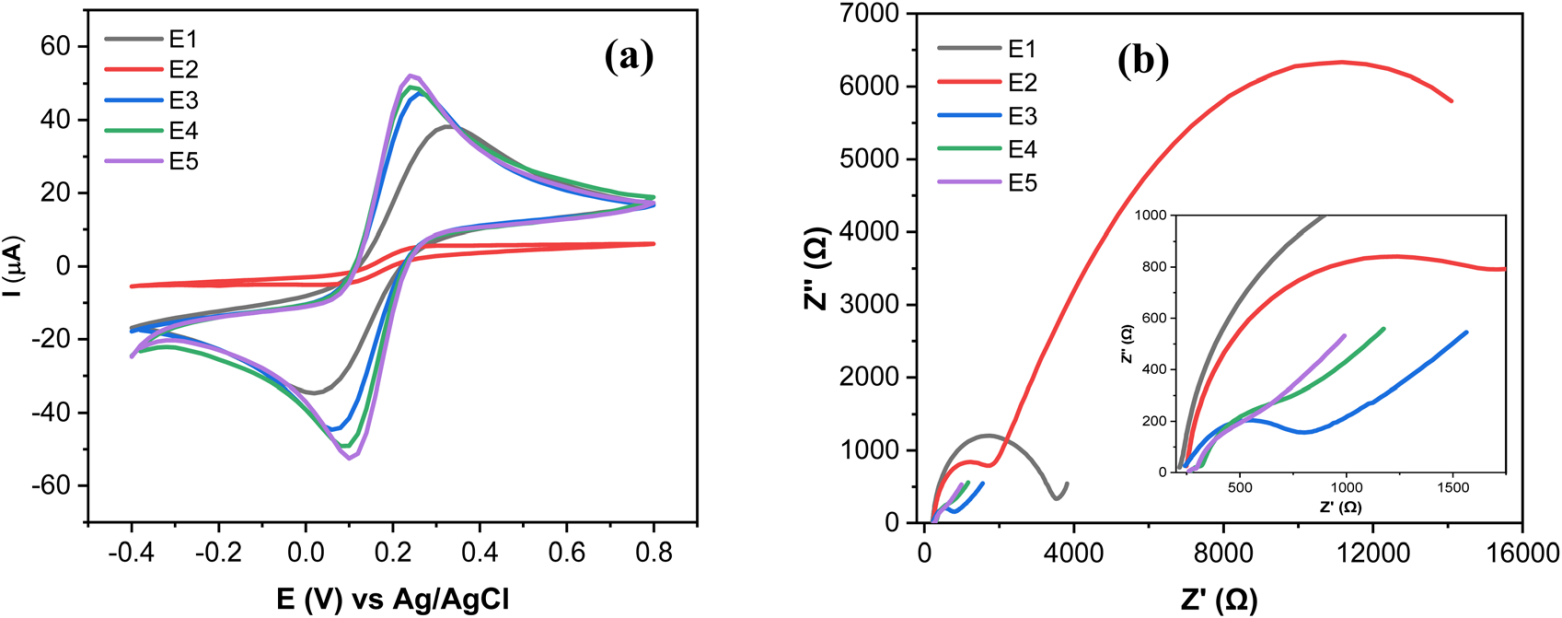
(a) CV curves at a scan rate of 25 mV s^−1^, and (b) Nyquist plots of 5.0 mmol L^−1^ [Fe(CN)6]^3−/4−^ in 0.1 mol L^−1^ KCl obtained with a bare SPCE (E1), GO@SPCE (E2), AgNPs@SPCE (E3), GO/IL@SPCE (E4), and AgNPs@GO/IL@SPCE (E5). Note: geometric area of the working electrode: 7 mm^2^.

The electrochemical characterization of SPCE (E1), GO@SPCE (E2), AgNPs@SPCE (E3), GO/IL@SPCE (E4), and AgNPs@GO/IL@SPCE (E5) was performed using CV and EIS in the presence of the redox probe [Fe(CN)6]^3−/4−^ as presented in Fig. 6a and b, respectively. The E5 exhibits the highest anodic and cathodic peak currents compared to other electrodes. The peak currents increased in the following trend: E2 < E1 < E3 < E4 < E5, where E2 has the lowest peak currents, which may be due to the high repulsion force between the GO surface and the negatively charged redox probe [Fe(CN)_6_]^3−/4−^.^36^ After modifying the E1 with AgNPs (E3), GO/IL (E4), and AgNPs@GO/IL (E5) step by step, the electron transfer rate significantly improved due to the increase in the electroactive surface area of the modified electrodes, thus enhancing the electrocatalytic activity. The electroactive surface area was evaluated by running the CV of the modified electrodes at different scan rates (Fig. S4–S8†) and using the Randles–Sevcik equation.^21^ The electroactive surface area changes in the following order: E2 (0.008 cm^2^) < E1 (0.058 cm^2^) <E3 (0.063 cm^2^) <E4 (0.071 cm^2^) <E5 (0.075 cm^2^).

**Fig. 7:**
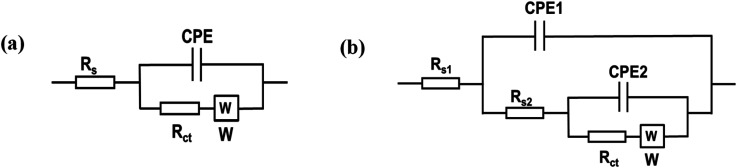
Equivalent circuits applied to model EIS data (a) for SPCE (E1) and AgNPs@SPCE (E3). (b) GO@SPCE (E2), GO/IL@SPCE (E4), and AgNPs@GO/IL@SPCE (E5).

Fig. 6b presents the Nyquist plots of the impedance spectra of E1–E5. The EIS data was fitted using modified Randles equivalent circuits, which include the electrolyte solution resistance (*R*_s_), constant phase element (CPE), Warburg impedance (*W*), and charge transfer resistance (*R*_ct_). The circuit shown in Fig. 7a is suitable for E1 and E3, while the circuit in Fig. 7b, with an additional constant phase, is suitable for E2, E4, and E5. We attribute this to the introduction of an additional interface resulting from the incorporation of GO, which has the effect of increasing the overall impedance through the addition of a non-ideal capacitive element. The *R*_ct_ values were in the order of E2 (18 200 Ω) > E1(3060 Ω) > E3 (632) Ω > E4 (442 Ω) > E5 (37 Ω). This demonstrates that AgNPs@GO/IL@SPCE (E5) has the lowest charge resistance, the highest conductivity, and the fastest electron transfer ability. The data obtained from both CV and EIS spectra show consistency.

### 3.5. Electrochemical reduction mechanism of MP

**Fig. 8:**
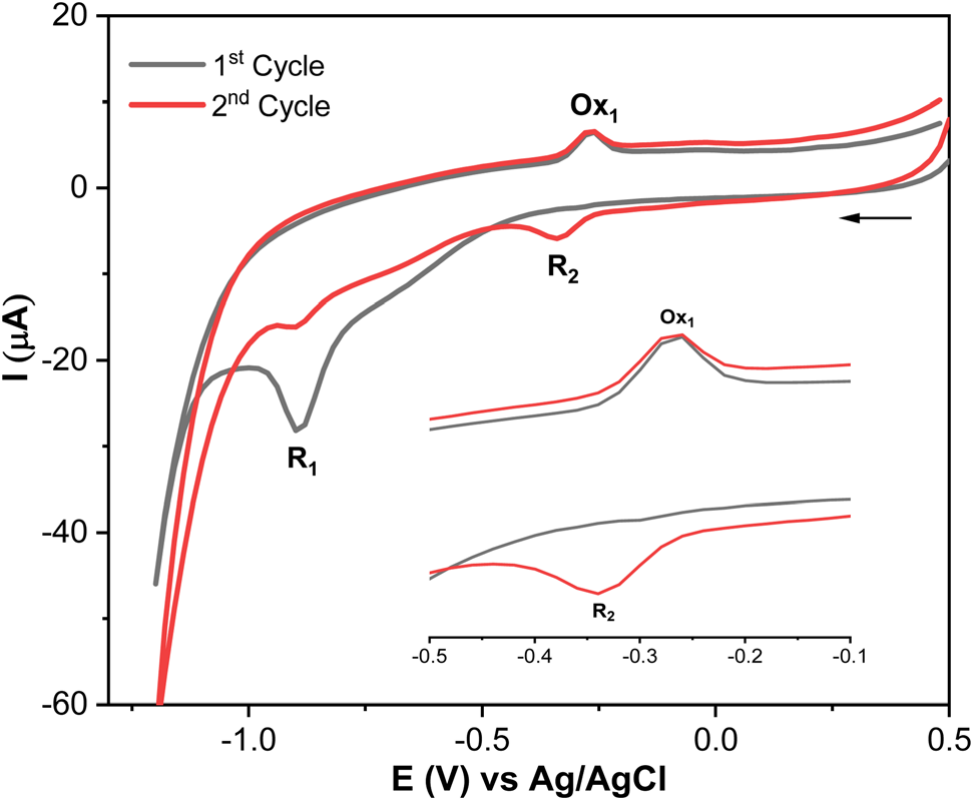
Electrochemical behavior of MP (20 μmol L^−1^; in 0.1 mol L^−1^ PBS pH 7; scan rate 100 mV s^−1^).

**Scheme 1:**
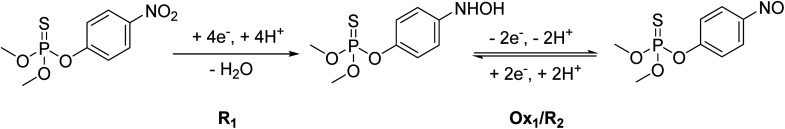
Electrochemical reaction mechanism of MP.^20^

Methyl parathion (MP) contains an electrochemically active group (–NO_2_), which exhibits multiple redox peaks.^41,42^ Therefore, understanding its electrochemical behavior is crucial, as it can help identify the most suitable peak for accurate MP quantification. Therefore, the electrochemical reduction mechanism of MP at AgNPs@GO/IL@SPCE was studied in 0.1 mol L^−1^ PBS (pH 7) as a supporting electrolyte by CV, as shown in Fig. 8 and Scheme 1 (additional results of other electrodes are presented in Fig. S9†).

MP displays a sharp irreversible peak at −0.9 V (1^st^ cycle, *R*_1_), which corresponds to the reduction of the nitro group (–NO_2_) to hydroxylamine group (–NHOH) by the exchange of four electrons and four protons (Scheme 1, *R*_1_). When the direction of the potential sweep is switched in the anodic scan, an oxidation peak at −0.27 V (1^st^ cycle, Ox_1_) was observed, which corresponds to the oxidation of the hydroxylamine group (–NHOH) to the nitroso group (–NO) through a two-electron and two-proton reaction process (Scheme 1). This reaction is reversible, producing its reduced form in the second cathodic sweep at −0.34 V (2^nd^ cycle, *R*_2_, Scheme 1). This electrochemical behavior of MP is consistent with that of other compounds containing the nitro functionality, such as nitrophenols,^43^ paraoxon,^44^ parathion-ethyl,^45^ and fenitrothion.^46^ These findings show that the AgNPs@GO/IL@SPCE electrode is a promising candidate for electrocatalytic reduction and determination of MP in real samples. Based on MP's electrochemical behavior, the next studies will focus on the irreversible reduction peak associated with nitro group reduction (*R*_1_) for kinetics analysis and MP quantification.

### 3.6. Effect of the pH and scan rate on the reduction of MP

**Fig. 9:**
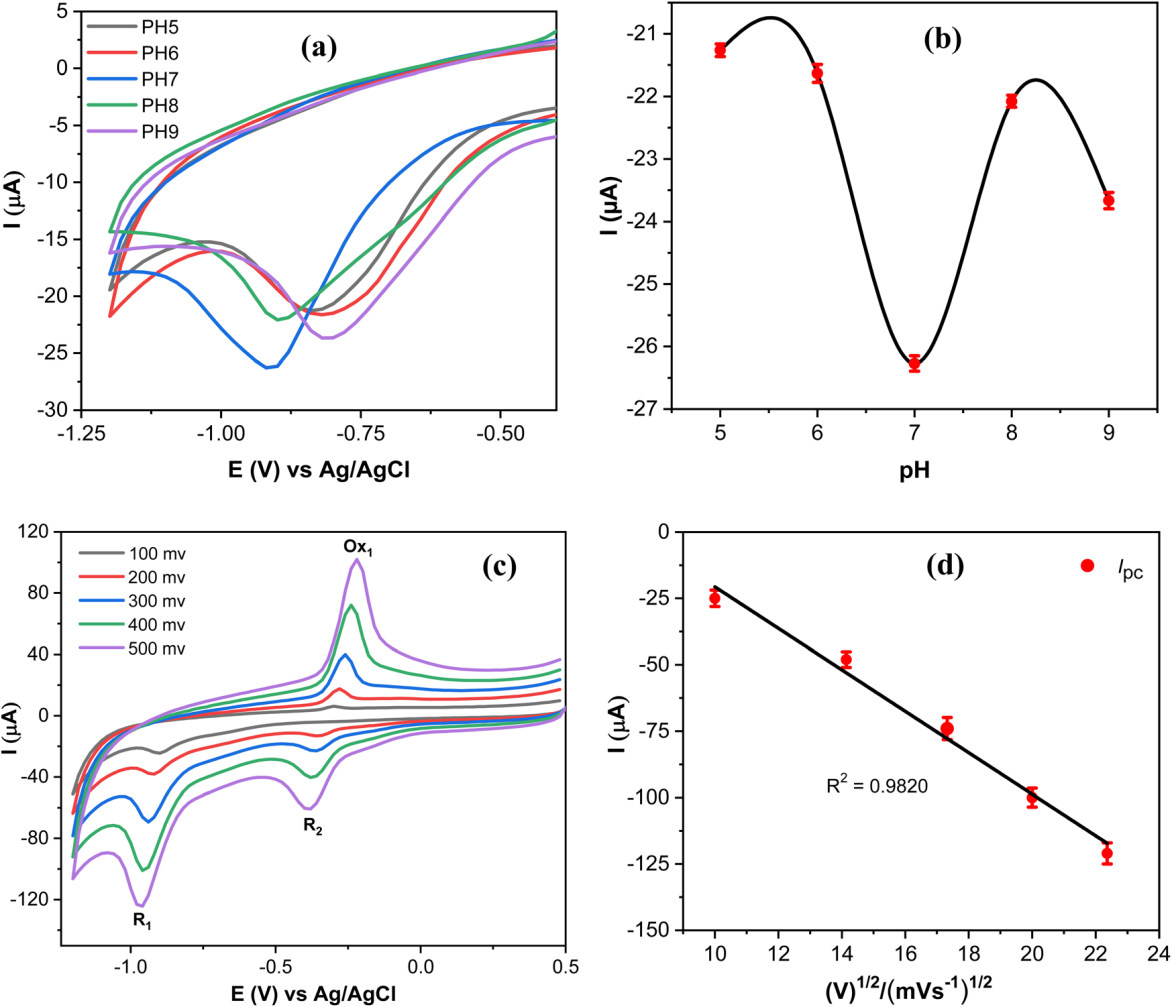
(a) CV curves obtained at AgNPs@GO/IL@SPCE in 0.1 mol L^−1^ PBS containing 20 μmol L^−1^ MP at different pH; scan rate 100 mV s^−1^. (b) The correlation between the pH and the reduction peak current of MP. (c) CV curves of 20 μmol L^−1^ PM in 0.1 mol L^−1^ PBS (pH 7) at different scan rates. (d) A graph depicting the peak currents of MP reduction (*R*_1_) plotted against the square root of the scan rate. Note: geometric area of the working electrode: 7 mm^2^.

Since the electrochemical reduction of MP involves proton transfer, the influence of pH values on the electrochemical response of MP was investigated to accurately determine the optimal catalytic activity of the proposed sensor as shown in Fig. 9a.

The reduction peak current of MP increased as the pH values increased and attained the maximum reduction current at pH 7, and then the reduction current decreased at higher values (Fig. 9b). Thus, supporting electrolytes with pH 7 were selected as an optimal medium throughout this work. The reduction potential of MP varies with the pH of the supporting electrolyte, due to the involvement of protons in the electrochemical reduction of MP,^46^ as presented in Scheme 1. The reaction mechanism of MP on the AgNPs@GO/IL@SPCE (E5) electrode can be determined by studying the peak current measurements as a function of the square root of the scan rate. Thus, CV_S_ of 20 μmol L^−1^ PM were measured at different scan rates, as depicted in Fig. 9c. It is observed that both cathodic and anodic peak currents gradually increase upon increasing the scan rate. Moreover, the peak currents of the *R*_1_, Ox_1_, and *R*_2_ processes are linearly dependent on the scan rate, with regression coefficients *R*^2^ = 0.9820, 0.9851, and 0.9873, respectively, as shown in Fig. 9d and S10.† This behavior indicates that the redox reaction of MP on the AgNPs@GO/IL@SPCE (E5) electrode follows a surface-controlled mechanism.^20^

### 3.7. Electrocatalytic performance of different modified electrodes

**Fig. 10:**
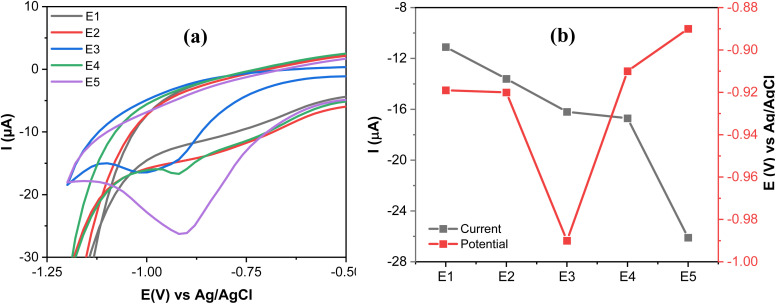
(a) CVs of SPCE (E1), GO@SPCE (E2), AgNPs@SPCE (E3), GO/IL@SPCE (E4) and AgNPs@GO/IL@SPCE (E5) in the presence of 20 μmol L^−1^ MP in 0.1 mol L^−1^ PBS (pH 7); scan rate 100 mV s^−1^. (b) The variation of *I*_pa_ and *E*_pa_ of MP reduction at different electrodes. Note: geometric area of the working electrode: 7 mm^2^.

The electrocatalytic performance of E1–E5 was studied through CV measurements of 20 μmol L^−1^ MP in 0.1 mol L^−1^ PBS at a scan rate of 100 mV s^−1^. Fig. 10a and b show a broad irreversible reduction peak at (−11.1 μA, −0.92 V) and at (−13.6 μA, −0.92 V) on E1 and E2, respectively. This may be due to the weak catalytic reactivity of E1 and E2. In contrast, the catalytic performance improved in E3 and E4, which can be revealed by a more pronounced reduction peak current of MP at (−16.2 μA, 0.99 V) and at (−16.7 μA, −0.91 V) on E3 and E4, respectively.

A comparison between E2 (GO) and E4 (IL/GO) demonstrates the significant role of IL in boosting electrocatalytic activity. This enhancement can be attributed to the augmented active surface area (E4 exhibits a ninefold increase compared to E2, as outlined in Section 3.4), which provides additional active sites for electrochemical reactions. Moreover, the high ionic conductivity of the IL increases the overall conductivity of the composite, thus facilitating more efficient charge transfer (Section 3.4). The reduction current and the reduction potential for MP are (−26.1 μA, −0.89 V) at E5. The results indicate that the E5 electrode caused a shift in the reduction potential of MP to a more positive value and an increase in the reduction current compared to the other electrodes. This may be attributed to the high electrocatalytic activity and strong adsorption capabilities of E5, along with its effective interaction with the MP, which is enhanced by the synergetic effect of GO, IL, and AgNPs.

### 3.8. Semiempirical quantum chemistry calculations

**Fig. 11:**
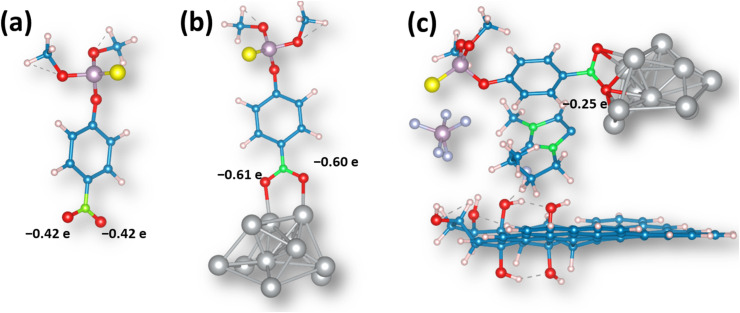
Optimized structures of (a) MP, (b) MP interacting with the Ag cluster, and (c) MP interactions with the Ag@GO/IL system.

As MP undergoes electrochemical reduction at the –NO_2_ group attached to the aromatic moiety,^47^ we have investigated the interaction of this group with the Ag cluster and analyzed how the addition of the GO sheet and IL affects the charge distribution and activation of the ML molecule. First, in the isolated MP molecule, the oxygen atoms in the –NO_2_ group bear a negative charge amounting to 0.42*e* (Fig. 11a). When the same group interacts with the Ag cluster, there is a net charge flow to the MP molecule, and in the optimized structure (Fig. 11b), the oxygen atoms in the –NO_2_ group bear the negative charge of ∼0.61*e*. This indicates that such bonded molecules are less prone to reduction.

When the (MP + Ag cluster) system landed on the GO sheet model, we did not observe an interaction between the Ag cluster and the GO sheet, and the distance between them was above 7.5 angstroms. However, once IL was added to the system, we observed the stabilization, and the IL acted as the “glue” in the system, stabilizing it *via* non-covalent interactions (Fig. S11†). However, the most important observation is that in the (MP + AgNPs@GO/IL) system, the oxygen atoms in the –NO_2_ group are electron deficient compared to those in the isolated MP molecule (Fig. 11c). This means that the –NO_2_ group of the MP molecule interacting with the AgNPs@GO/IL system is more prone to reduction. Thus, the IL component has a crucial role in stabilizing the interactions between Ag clusters and GO, allowing proper activation of the methyl-parathion molecule for electrochemical conversion.

### 3.9. Electrochemical determination of MP

**Fig. 12:**
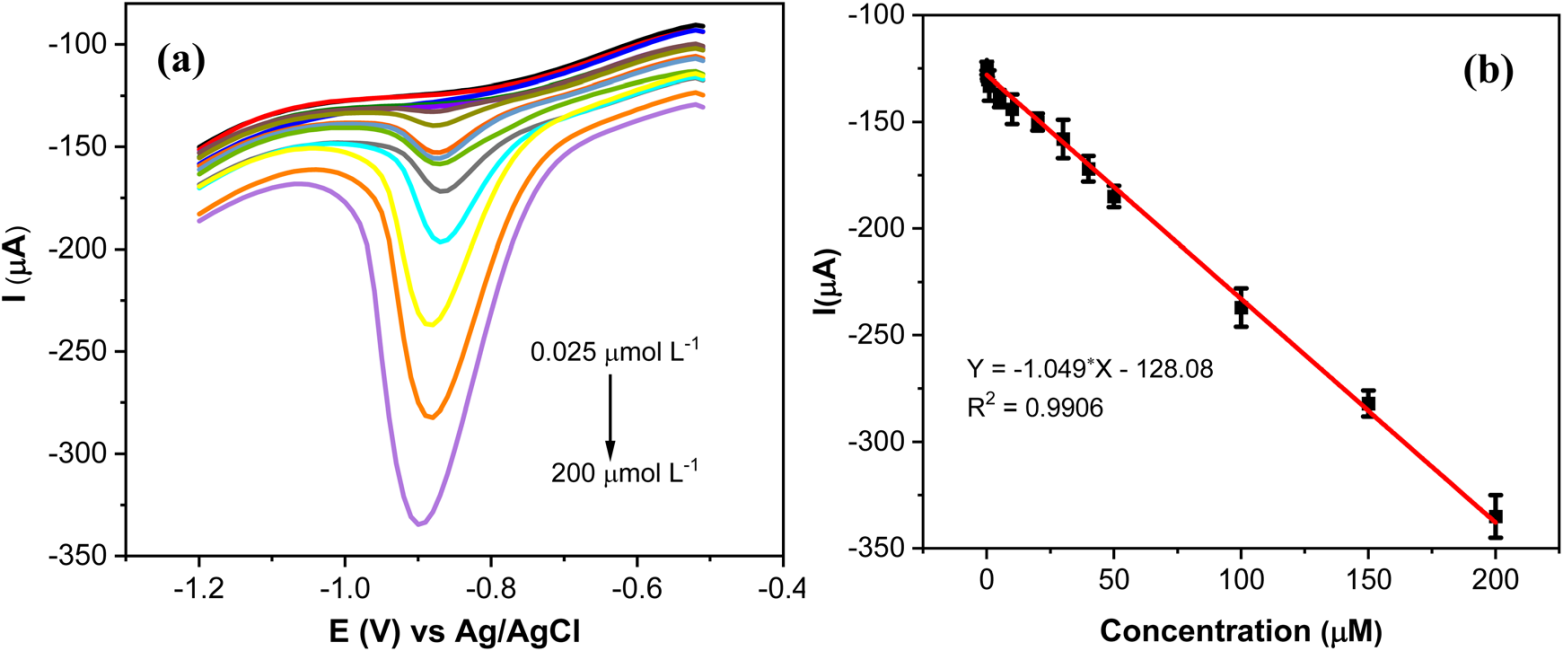
(a) SWV curves of AgNPs@GO/IL@SPCE (E5) in 0.1 mol L^−1^ PBS (pH 7) containing different concentrations of MP (0.025–200 μmol L^−1^); potential range (−0.5 to −1.2 V), amplitude 0.1 V; frequency 20 Hz. (b) The calibration plots of the peak currents *vs.* concentration of MP. Note: geometric area of the working electrode: 7 mm^2^.

The analytical performance of AgNPs@GO/IL@SPCE for the quantitative determination of MP was studied by SWV under optimal conditions (Fig. 12a). It is evident that the reduction peak current exhibits a discernible increase by increasing the concentration of MP from 0.025 to 200 μmol L^−1^. The slight cathodic shift in the reduction potential of MP with increasing concentration (Fig. 12a) is likely caused by the adsorption and accumulation of MP on the electrode surface, which hinders electron transfer and leads to the observed shift.

The peak current response has a linear relationship with the concentration of MP with a correlation coefficient of *R*^2^ = 0.9906 (Fig. 12b). The LoD of AgNPs@GO/IL@SPCE was calculated to be 0.009 μmol L^−1^ according to standard procedures mentioned in the relevant literature.^37^

The linear range and the detection limit were the main factors considered while assessing the developed sensor's analytical performance. Compared to other electrodes previously described for MP detection, our sensor displayed a straightforward, easy, and low-cost production procedure with a wide range of concentrations (Table 3). This suggests a promising sensing platform using AgNPs@GO/IL@SPCE to determine MP in the water environments.

**Table 3:** Comparison of different electrochemical sensors reported for MP determination^a^

Modified electrode	Technique	Linear range (μmol L^−1^)	LoD (μmol L^−1^)	References
Hal-MWCNTs/GCE	DPV	0.5–11	0.034	48
Sn/MoC@NC/GCE	DPV	0.2–39	0.035	49
MGPCS/GCE	DPV	0.1–15	0.011	50
MWCNT/zirconia	DPV	1.9–176	0.009	51
COOH–CNT/CPE	DPV	0.06–30	0.027	52
β-TCP/GCE	SWV	0.15–141	0.088	53
Z-NiO/CoO@CC	DPV	0.04–40	0.014	54
NiO@SPE	DPV	0.1–30	0.024	55
Ag/GNPs/ZrO_2_	SWV	1.0–50	0.1	56
ERGO–CS/Hb/FTO	SWV	0.076–0.988	0.079	57
GR-MWCNT@CeO_2_/GCE	DPV	0.01–10	0.028	58
MnCoP-core–shell/GCE	DPV	0.5–400	0.05	59
SCN@UIO-66/GCE	DPV	0.01–10	0.008	60
**AgNPs@GO/IL@SPCE**	**SWV**	**0.025–200**	**0.009**	**This work**

a Hal-MWCNTs: halloysite nanotubes/multi-walled; GCE: glassy carbon electrode; MoC: molybdenum carbide, NC: N-doped carbon; MGPCS: morning glory-like porous carbon nanosheets; COOH–CNT: carboxyl-group functionalized carbon nanotubes; CPE: carbon paper electrode; β-TCP: β-tricalcium phosphate (Ca_3_(PO_4_)_2_) nanoparticles; SPE: screen printed carbon electrode; GNPs: graphene nanoplatelets; ERGO–CS: electrochemically reduced graphene oxide–chitosan; Hb: hemoglobin; FTO: fluorine-tin oxide glass; GR-MWCNT: graphitized multiwalled carbon nanotubes; CeO_2_: cerium oxide; MnCoP: manganese cobalt phosphide; SCN: single-wall carbon nanotube networks; UIO-66: Zr-based metal–organic framework nanoparticles.

### 3.10. Selectivity, reproducibility, and stability studies

The design and evaluation of electrochemical sensing require careful consideration and optimization of other factors such as selectivity, repeatability, and stability. These elements determine the efficiency and reliability of electrochemical sensing in various scientific and practical applications. The selectivity of the AgNPs@GO/IL@SPCE (E5) electrode was tested using the SWV technique in the presence of potential interference. The SWV of 0.1 mol L^−1^ PBS (pH 7) containing 20 μmol L^−1^ PM was recorded in the presence of 100-fold inorganic ions (SO_4_^2−^, CO_3_^2−^, K^+^, Cl^−^, NO_2_^−^) and 10-fold concentrations of other pesticides (glyphosate; GLY, chlorpyrifos; CHL, pirimicarb; PMC, malathion; MLT).

**Fig. 13:**
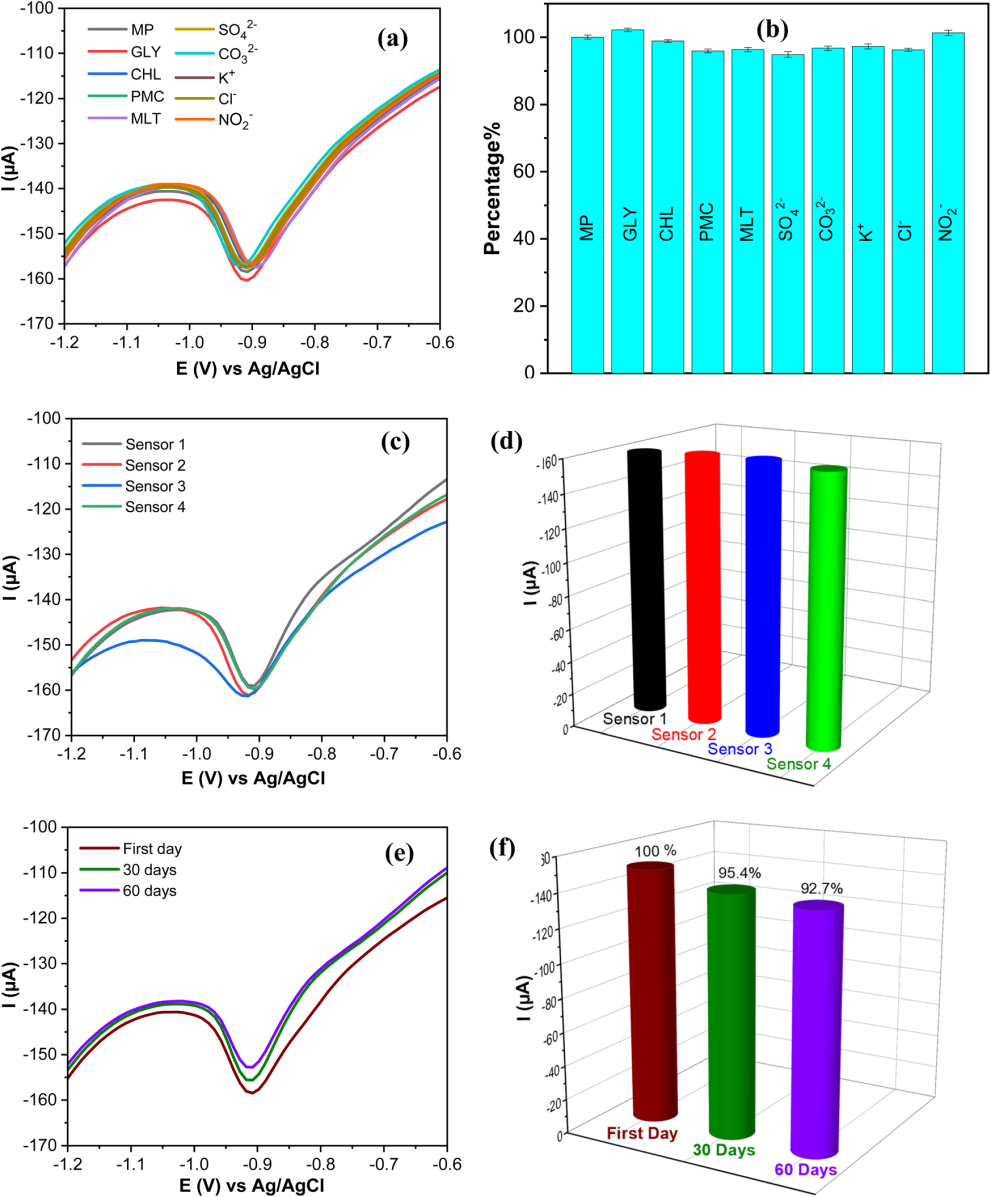
(a) SWV curves of 20 μmol L^−1^ PM on AgNPs@GO/IL@SPCE (E5) in the presence other interfering compounds. (b) Bare diagram of the current percentage change of PM reduction in the presence of interfering species. (c) SWV response of four AgNPs@GO/IL@SPCE (E5) electrodes towards 20 μmol L^−1^ PM. (d) Bare diagram evaluation of the reproducibility study. (e) SWV response of AgNPs@GO/IL@SPCE (E5) over storage time. (f) Bare diagram evaluation of the long-term stability study. Note: geometric area of the working electrode: 7 mm^2^.

As depicted in Fig. 13a and the relative error bare diagram presented in Fig. 13b, the interferents do not significantly affect the reduction peak current (*R*_2_) of MP, indicating that our sensor has strong anti-interfering and reliable selectivity in complex matrices.

The reproducibility of the AgNPs@GO/IL@SPCE (E5) electrode was assessed by measuring the reduction peak current of MP on four independent modified electrodes prepared under the same optimal conditions described in Section 2.5 as shown in Fig. 13c and the corresponding bare diagram (Fig. 13d). The relative standard deviation (RSD) of the four electrodes was found to be 2.1%, suggesting that the sensor has a high degree of reproducibility. Additionally, the long-term stability of AgNPs@GO/IL@SPCE (E5) was evaluated by storing it at room temperature for 60 days and studying its current response. The results, shown in Fig. 12f and 13e, indicate that after 60 days, the sensor retained approximately 92.7% of its original current response, demonstrating the excellent stability of the modified electrode. This stability may be attributed to the significant interaction between the modifier materials, as discussed in Section 3.8.

### 3.11. Application to real samples

**Fig. 14:**
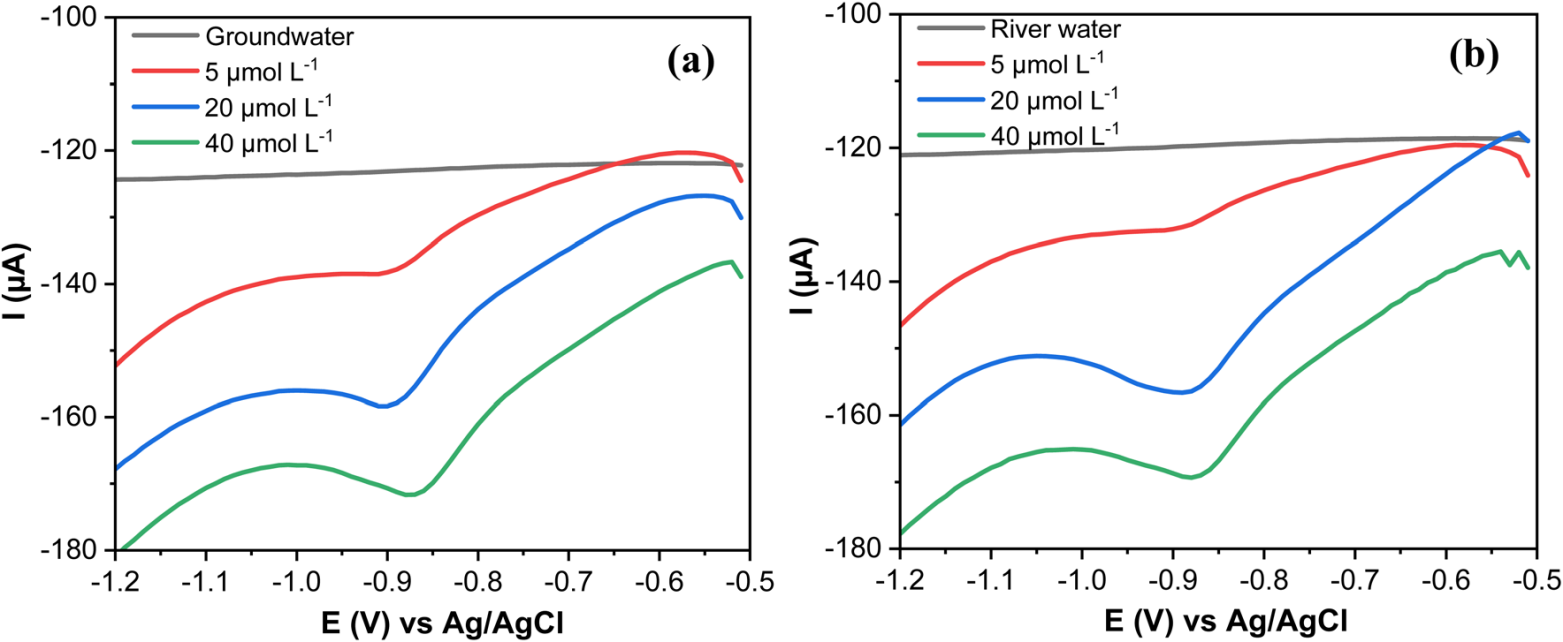
(a) SWV curves of MP spiked with groundwater samples and (b) river water. Note: geometric area of the working electrode: 7 mm^2^.

The quantification of MP in spiked groundwater and river water was performed to evaluate the suitability of the designed electrode AgNPs@GO/IL@SPCE (E5) for real sample applications. The SWV response of the groundwater and surface water spiked with known concentrations of MP was measured, as displayed in Fig. 14a and b.

The recovery values (96.80–99.68%) and the RSD (1.57–2.46) of MP spiked in groundwater samples are summarized in Table 4. The recoveries are (95.60–99.35%) and RSD (2.17–3.26) for MP spiked in river water (Table 4). The obtained values show that the AgNPs@GO/IL@SPCE (E5) is highly feasible for the practical determination of MP in real samples.

**Table 4:** Determination of MP in the groundwater and surface water using AgNPs@GO/IL@SPCE (E5)

Samples	Added (μmol L^−1^)	Found (μmol L^−1^)	Recovery (%)	RSD (%) (*n* = 3)
Groundwater	5	4.84	96.80	2.46
	20	19.93	99.68	1.57
	40	39.22	98.07	2.32
River water	5	4.78	95.60	3.26
	20	19.76	98.80	3.02
	40	39.74	99.35	2.17

## 4. Conclusion

The results of this study demonstrate the successful development of effective sensors for the electrochemical detection of MP using AgNPs@GO/IL modified SPCE. The synthesis of quasi-spherical AgNPs by a straightforward wet-chemical method was confirmed by optical and morphological analysis. Integrating the synthesized AgNPs with the GO/IL composite resulted in the formation of a highly efficient electrocatalyst material. The comprehensive characterization of the electrocatalyst material, AgNPs@GO/IL, confirmed its structural integrity and highlighted the role of its components in enhancing electrocatalytic performance. Furthermore, semiempirical quantum chemistry calculations provided valuable insights into the electrochemical reduction mechanisms of MP on the electrode surface and the pivotal role of the IL component in stabilizing Ag clusters on the GO surface.

In addition, the exceptional analytical performance of the AgNPs@GO/IL@SPCE sensor, including its low LoD, wide linear range, and robust long-term stability, indicates that it may have significant potential for practical applications in environmental monitoring and pesticide residue analysis. Moreover, the AgNPs@GO/IL@SPCE sensor has demonstrated satisfactory results for determining MP in groundwater and surface water, which supports the feasibility of the proposed electrocatalyst material for quantifying MP in real-world applications.

## Data availability

The data associated with the manuscript are available in the ESI file.†

## Conflicts of interest

There are no conflicts to declare.

## Supplementary Material

NA-007-D4NA00919C-s001

## References

[cit1] Richardson R. J. and Makhaeva G. F., Organophosphorus Compounds, Elsevier, 3rd edn, 2014, vol. 3

[cit2] Kaushal J., Khatri M., Arya S. K. (2021). Ecotoxicol. Environ. Saf..

[cit3] Jokanovi M., Oleksak P., Kuca K. (2023). Toxicology.

[cit4] Gao N., He C., Ma M., Cai Z., Zhou Y., Chang G., Wang X., He Y. (2019). Anal. Chim. Acta.

[cit5] Shanmugam R., Manavalan S., Chen S., Keerthi M., Lin L. (2020). ACS Sustain. Chem. Eng..

[cit6] Guidelines for drinking-water quality: fourth edition incorporating the first and second addenda, WHO, 2022, https://www.who.int/publications/i/item/9789240045064 35417116

[cit7] Mali H., Shah C., Raghunandan B. H., Prajapati A. S., Patel D. H., Trivedi U., Subramanian R. B. (2023). J. Environ. Sci..

[cit8] Karimi-Maleh H., Darabi R., Baghayeri M., Karimi F., Fu L., Rouhi J., Niculina D. E., Gündüz E. S., Dragoi E. N. (2023). J. Food Meas. Charact..

[cit9] da Silva D. F., Paiva Silva F. E., Silva F. G. S., Nunes G. S., Badea M. (2015). Pest Manage. Sci..

[cit10] Tümay Özer E., Osman B., Parlak B. (2020). Microchem. J..

[cit11] Chen L., Dang X., Ai Y., Chen H. (2018). J. Sep. Sci..

[cit12] García-Córcoles M. T., Rodríguez-Gómez R., de Alarcón-Gómez B., Çipa M., Martín-Pozo L., Kauffmann J. M., Zafra-Gómez A. (2019). Crit. Rev. Anal. Chem..

[cit13] Baranwal J., Barse B., Gatto G., Broncova G., Kumar A. (2022). Chemosensors.

[cit14] Wu B., Xiao L., Zhang M., Yang C., Li Q., Li G., He Q., Liu J. (2021). J. Solid State Chem..

[cit15] Zhang S., Ling P., Chen Y., Liu J., Yang C. (2023). Diamond Relat. Mater..

[cit16] Govindasamy M., Mani V., Chen S. M., Chen T. W., Sundramoorthy A. K. (2017). Sci. Rep..

[cit17] Sooraj M. P., Mathew B. (2019). Food Anal. Methods.

[cit18] Rahmani T., Hajian A., Afkhami A., Bagheri H. (2018). New J. Chem..

[cit19] Govindasamy M., Sakthinathan S., Chen S. M., Chiu T. W., Sathiyan A., Merlin J. P. (2017). Electroanalysis.

[cit20] Hou X., Liu X., Li Z., Zhang J., Du G., Ran X., Yang L. (2019). New J. Chem..

[cit21] Sen S., Roy A., Sanyal A., Devi P. S. (2022). Beilstein J. Nanotechnol..

[cit22] Rodrigues G. H. S., Miyazaki C. M., Rubira R. J. G., Constantino C. J. L., Ferreira M. (2019). ACS Appl. Nano Mater..

[cit23] Yao J., Liu Z., Jin M., Zou Y., Chen J., Xie P., Wang X., Akinoglu E. M., Zhou G., Shui L. (2020). Sens. Actuators, B.

[cit24] Nehru R., Hsu Y. F., Wang S. F., Di Dong C., Govindasamy M., Habila M. A., AlMasoud N. (2021). Microchim. Acta.

[cit25] He T., Dai Q., Huang W., Wang X. (2018). Appl. Phys. A: Mater. Sci. Process..

[cit26] Kargar S., Elhamifar D., Zarnegaryan A. (2021). Surf. Interfaces.

[cit27] Bouazizi N., Vieillard J., Bargougui R., Couvrat N., Thoumire O., Morin S., Ladam G., Mofaddel N., Brun N., Azzouz A., Le Derf F. (2019). J. Alloys Compd..

[cit28] Pryshchepa O., Pomastowski P., Buszewski B. (2020). Adv. Colloid Interface Sci..

[cit29] Khatoon U. T., Nageswara Rao G. V. S., Mohan K. M., Ramanaviciene A., Ramanavicius A. (2017). Vacuum.

[cit30] Stewart J. J. P. , Stewart computational chemistry – MOPAC, 2016, https://openmopac.net/

[cit31] Stewart J. J. P. (2013). J. Mol. Model..

[cit32] Depizzol D. B., Paiva M. H. M., Dos Santos T. O., Gaudio A. C. (2005). J. Comput. Chem..

[cit33] Jmol: an open-source Java viewer for chemical structures in 3D, https://www.jmol.org/

[cit34] Momma K., Izumi F. (2008). J. Appl. Crystallogr..

[cit35] Klamt A., Schüürmann G. (1993). J. Chem. Soc., Perkin Trans. 2.

[cit36] Weheabby S., You S., Pašti I. A., Al-Hamry A., Kanoun O. (2024). Measurement.

[cit37] Weheabby S., Wu Z., Al-Hamry A., Pašti I. A., Anurag A., Dentel D., Tegenkamp C., Kanoun O. (2023). Microchem. J..

[cit38] Elmahdy M. M., Fahmy T., Aldhafeeri K. A., Ibnouf E. O. (2021). Mater. Chem. Phys..

[cit39] Zhang D., Peng L., Shi N., Yu Y., Min Y., Epstein A. J. (2017). J. Mater. Sci..

[cit40] Das M., Aswathy T. R., Pal S., Naskar K. (2021). Eur. Polym. J..

[cit41] Serrà A., Artal R., Pozo M., Garcia-Amorós J., Gómez E. (2020). Catalysts.

[cit42] Silvester D. S., Wain A. J., Aldous L., Hardacre C., Compton R. G. (2006). J. Electroanal. Chem..

[cit43] Hryniewicz B. M., Orth E. S., Vidotti M. (2018). Sens. Actuators, B.

[cit44] Kumar P., Ba D., Chen S., Hunsur C., Surareungchai W. (2022). Chemosphere.

[cit45] Kokulnathan T., Wang T., Ahmed F. (2021). J. Environ. Chem. Eng..

[cit46] Kokulnathan T., Wang T., Duraisamy N., Ashok E. (2021). J. Hazard. Mater..

[cit47] Xia S., Zhang J., Li C. (2010). Anal. Bioanal. Chem..

[cit48] Zhao H., Ma H., Li X., Liu B., Liu R., Komarneni S. (2020). Appl. Clay Sci..

[cit49] Li R., Shang M., Zhe T., Li M., Bai F., Xu Z., Bu T., Li F., Wang L. (2023). J. Hazard. Mater..

[cit50] Wan X., Wang Q., Guo X., Chen L., Hongyuan T. E. (2023). J. Porous Mater..

[cit51] Caetano K. dos S., da Rosa D. S., Pizzolato T. M., dos Santos P. A. M., Hinrichs R., Benvenutti E. V., Dias S. L. P., Arenas L. T., Costa T. M. H. (2020). Microporous Mesoporous Mater..

[cit52] Wang C., Zhong J., Hu J., Zhang G. (2021). Int. J. Electrochem. Sci..

[cit53] Sudhan N., Sekar C. (2021). Front. Nanotechnol..

[cit54] Xu Z., Li R., Zhao S., Zhangsun H., Wang Q., Wang L. (2021). Chem. Eng. J..

[cit55] Khairy M., Ayoub H. A., Banks C. E. (2018). Food Chem..

[cit56] Ulloa A. M., Glassmaker N., Oduncu M. R., Xu P., Wei A., Cakmak M., Stanciu L. (2021). ACS Appl. Mater. Interfaces.

[cit57] Kaur R., Rana S., Lalit K., Singh P., Kaur K. (2020). Biosens. Bioelectron..

[cit58] Wang Z., Liu Y., Li F., Dubovyk V., Guo M., Zhu G., Ran Q., Zhao H. (2022). J. Mater. Res. Technol..

[cit59] Karuppusamy N., Jeyaraman A., Chen T. W., Chen S. M., Packiaraj D. D. F., Al-Mohaimeed A. M., Al-onazi W. A., Elshikh M. S., Yu J. (2024). Food Chem..

[cit60] Han J., Li F., Zhao M., Guo M., Liu Y., Guo X., Ran Q., Wang Z., Zhao H. (2024). Microchem. J..

