# Supplementary material for: Enhanced electrochemical sensing of methyl parathion using AgNPs@IL/GO nanocomposites in aqueous matrices
Source: Nanoscale Adv. 2025 Feb 24;7(8):2195–208. doi: 10.1039/d4na00919c (PMC11848744; doi:10.1039/d4na00919c)
Supplement: NA-007-D4NA00919C-s001 [file NA-007-D4NA00919C-s001.pdf]

Supplementary Information (SI) for Nanoscale Advances.
This journal is © The Royal Society of Chemistry 2025

Supplementary Information

Enhanced electrochemical sensing of methyl parathion using AgNPs@IL/GO nanocomposites in aqueous matrices

Saddam Weheabby [a*], Ziyuan Liu [a], Igor A. Pašti [b], Vladimir Rajić [c], Marcio Vidotti [d], Olfa Kanoun [a]

[a] Chemnitz University of Technology, Measurement and Sensor Technology, 09126 Chemnitz, Germany

[b] University of Belgrade – Faculty of Physical Chemistry, Studentski trg 12-16, 11158 Belgrade, Serbia

[c] University of Belgrade, VINČA Institute of Nuclear Sciences - National Institute of the Republic of Serbia, Mike Petrovica Alasa 12-14, 11000 Belgrade, Serbia

[d] Grupo de Pesquisa em Macromoléculas e Interfaces, Universidade Federal do Paraná (UFPR), CxP 19032 - Curitiba 81531-980, PR, Brazil

[*]Corresponding author: saddam.weheabby@etit.tu-chemnitz.de

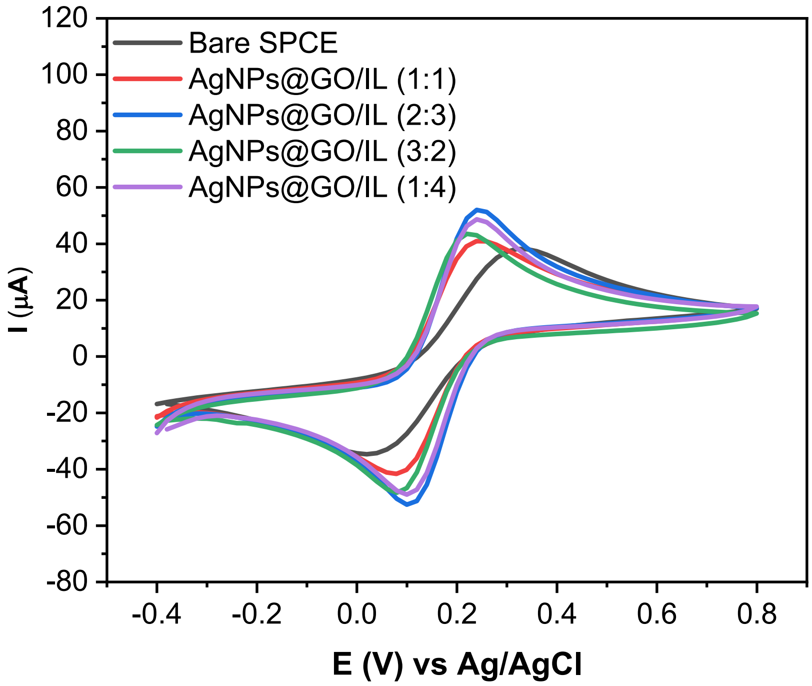

Figure S1. Optimization AgNP@GO/IL composite ratio in presence of 5.0 mmol L^{-1} $[Fe(CN)6]^{3-/4-}$ in 0.1 M KCl; scane rate 25 mV.

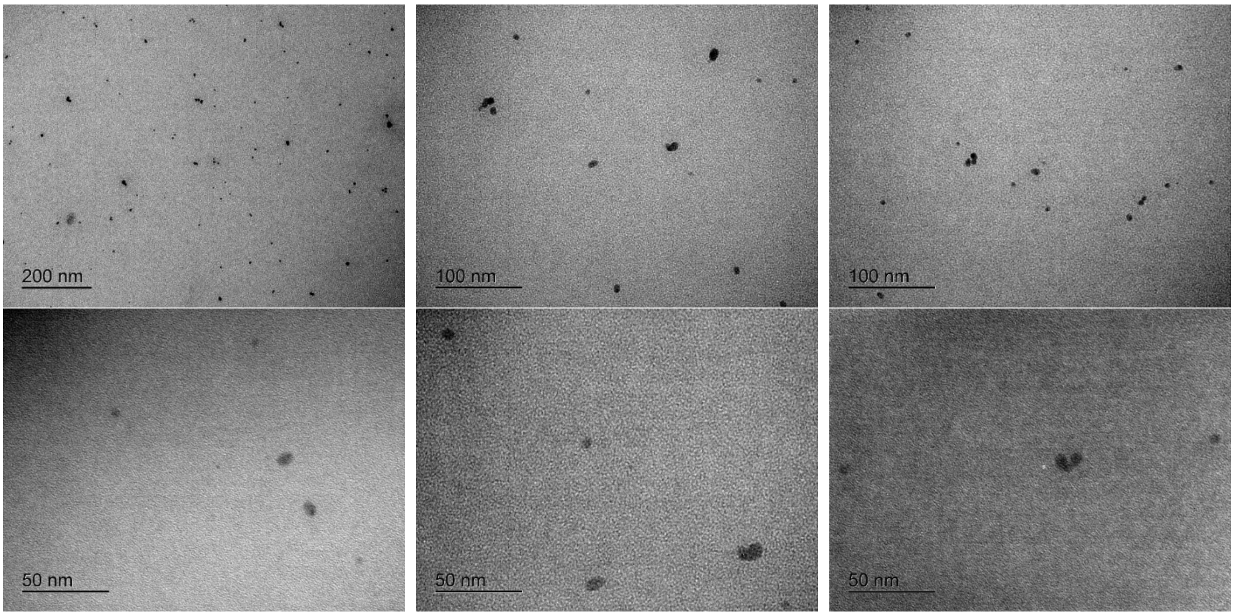

Figure S2. Representative TEM images of AgNPs obtained from different synthesis and different regions of the grid.

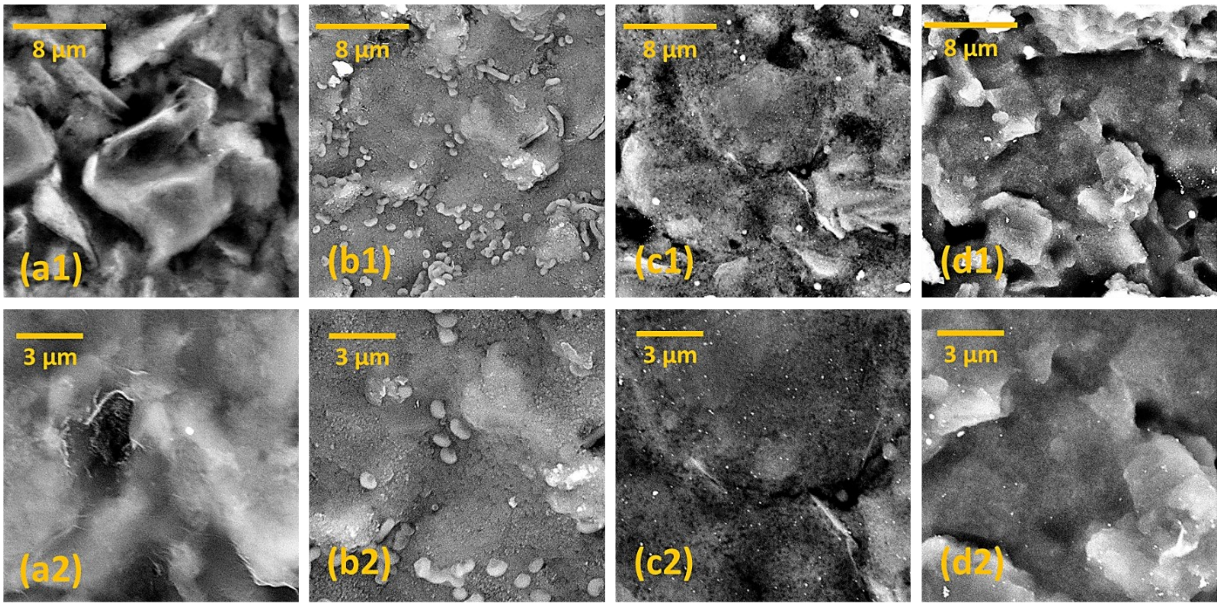

Figure S3. SEM images at magnifications ×10,000 (marked 1) and ×20,000 (marked 2), of the (a) GO@SPCE, (b) GO/IL@SPCE, (c) AgNPs@SPCE, and (d) AgNPs@GO/IL@SPCE. GO@SPCE and GO/IL@SPCE are covered with a Cu layer using a plasma coating procedure.

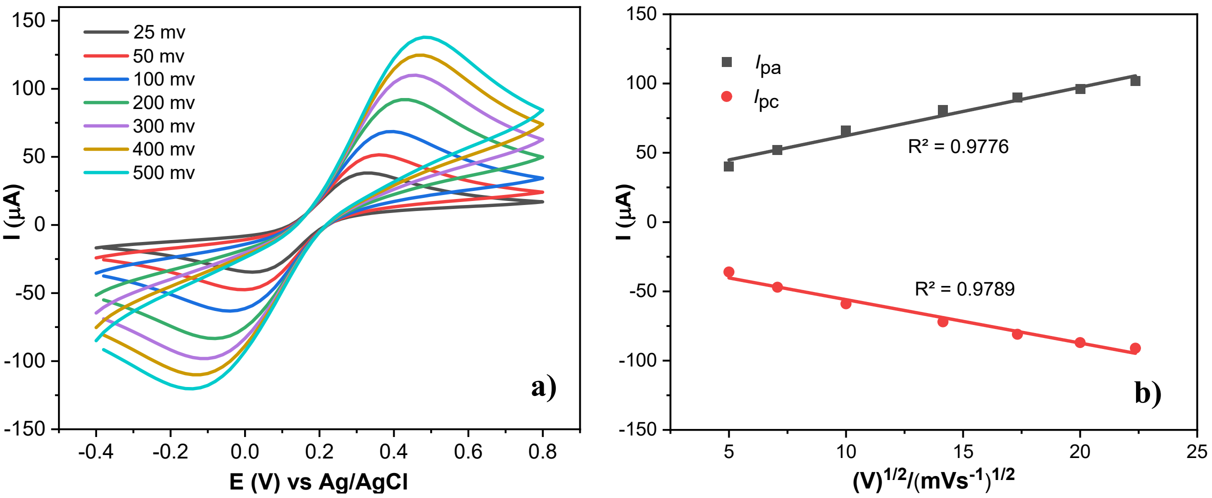

Figure S4. a) Cyclic voltammograms of 5.0 mmol L^{-1} $[Fe(CN)6]^{3-/4-}$ in 0.1 M KCl obtained with a bare SPCE at different scan rates. b) Anodic current and cathodic current vs. sq. root of scan rates.

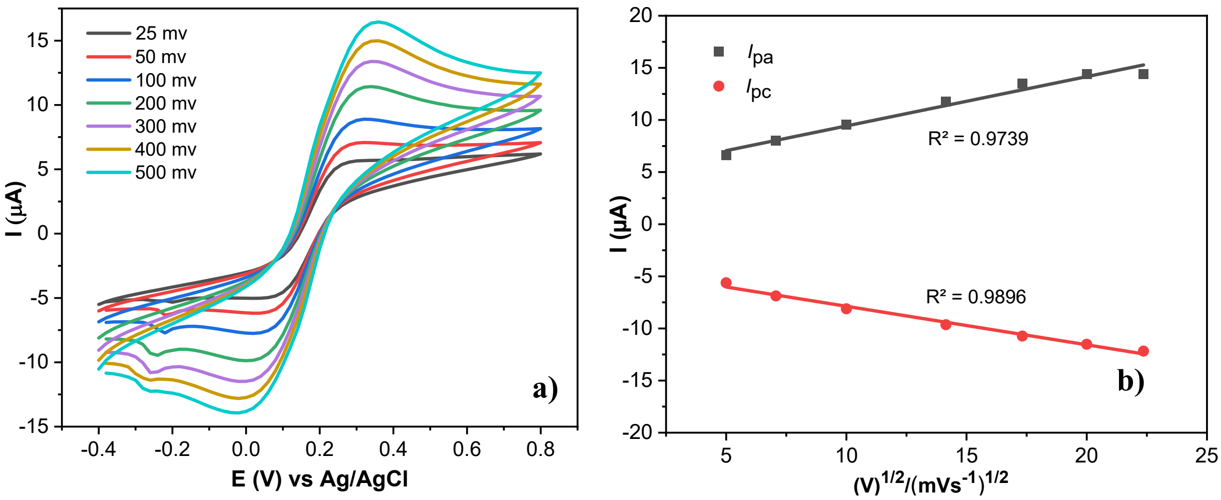

Figure S5. a) Cyclic voltammograms of 5.0 mmol L^{-1} $[Fe(CN)6]^{3-/4-}$ in 0.1 M KCl obtained with GO@SPCE different scan rates. b) Anodic current and cathodic current vs. sq. root of scan rates.

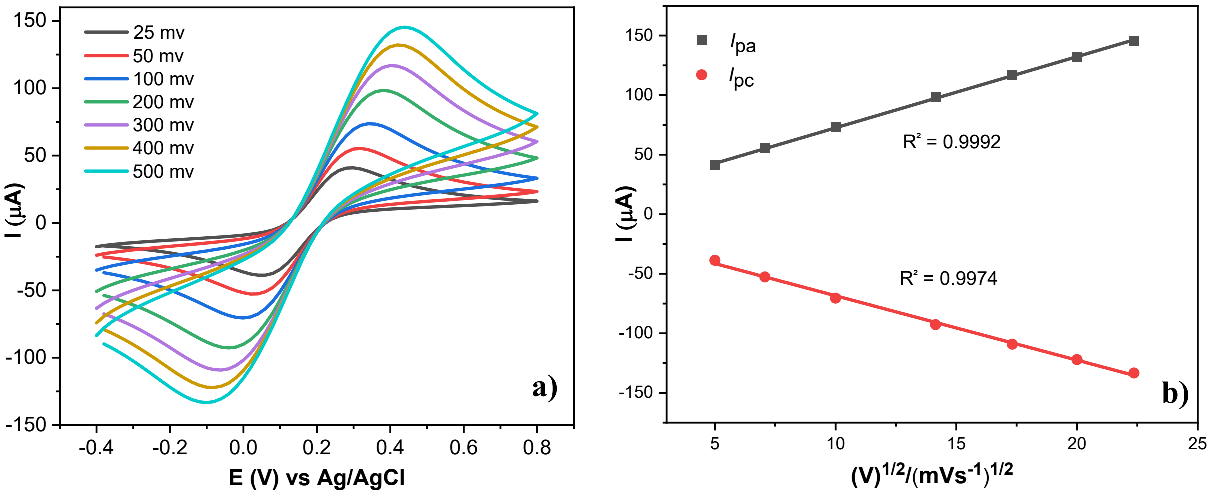

Figure S6. a) Cyclic voltammograms of 5.0 mmol L^{-1} $[Fe(CN)6]^{3-/4-}$ in 0.1 M KCl obtained with AgNPs@SPCE at different scan rate. b) Anodic current and cathodic current vs. sq. root of scan rates.

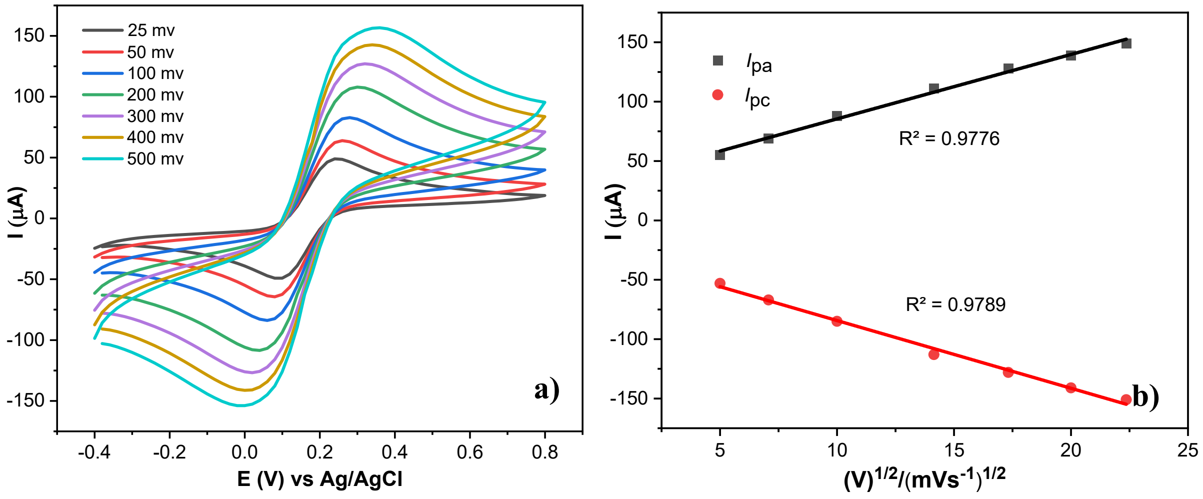

Figure S7. a) Cyclic voltammograms of 5.0 mmol L^{-1} $[Fe(CN)6]^{3-/4-}$ in 0.1 M KCl obtained with GO/IL@SPCE at different scan rate. b) Anodic current and cathodic current vs. sq. root of scan rates

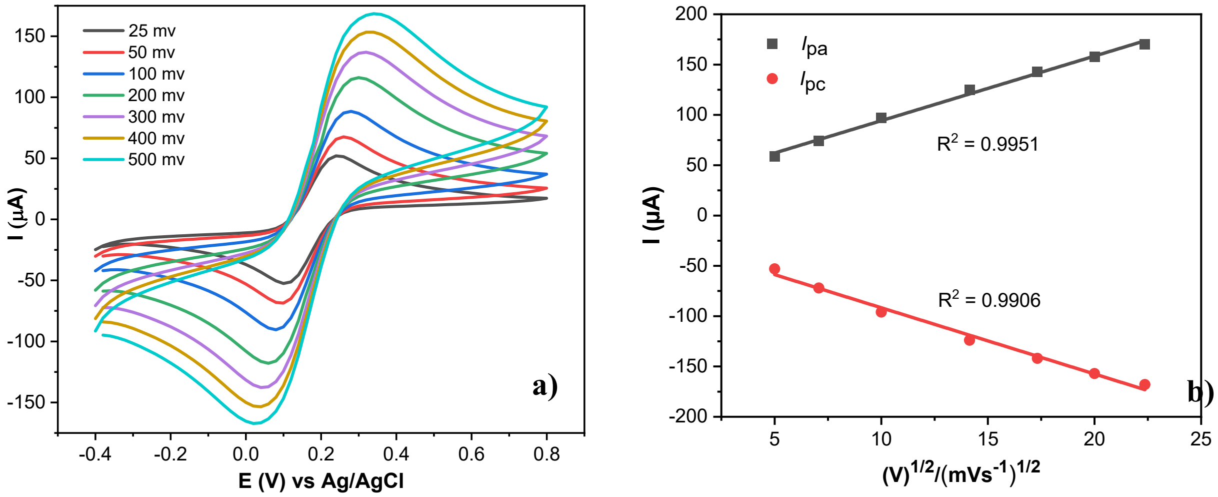

Figure S8. a) Cyclic voltammograms of 5.0 mmol L^{-1} $[Fe(CN)6]^{3-/4-}$ in 0.1 M KCl obtained with AgNPs@GO/IL@SPCE at different scan rate. b) Anodic current and cathodic current vs. sq. root of scan rates.

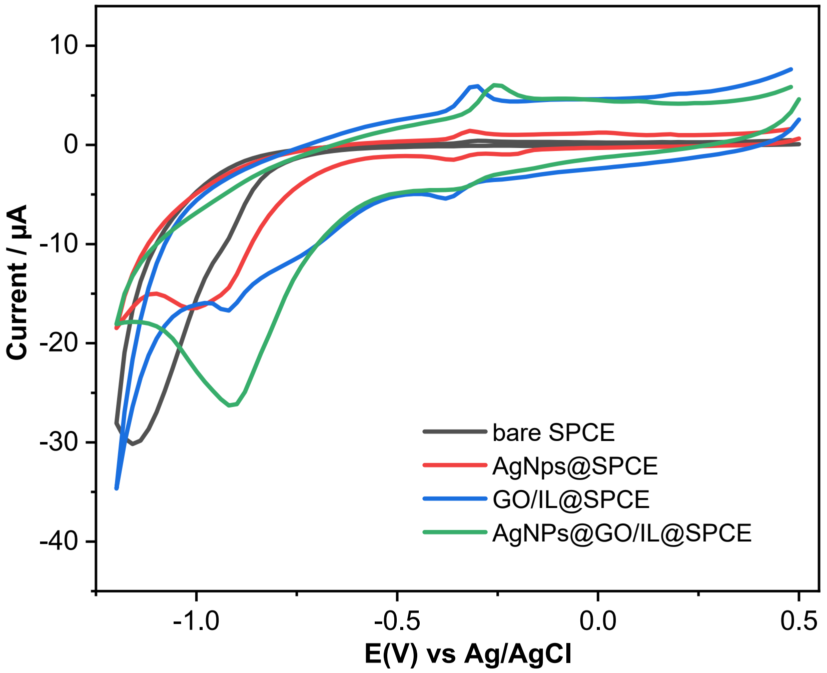

Figure S9. Electrochemical behavior of MP at different eletodes (20 µmol L^{-1} MP; in 0.1 M PBS pH 7; Scan rate 100 mV^{s-1})

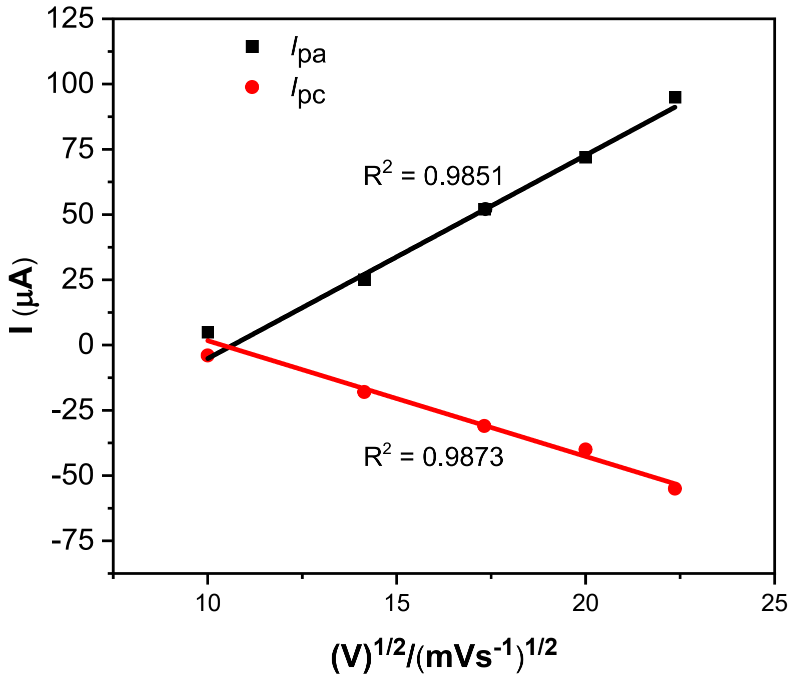

Figure S10. Anodic current and cathodic current vs. sq. root of scan rates of MP at AgNPs@GO/IL@SPCE (E5) electrode (Ox_1 and R_2 processes).

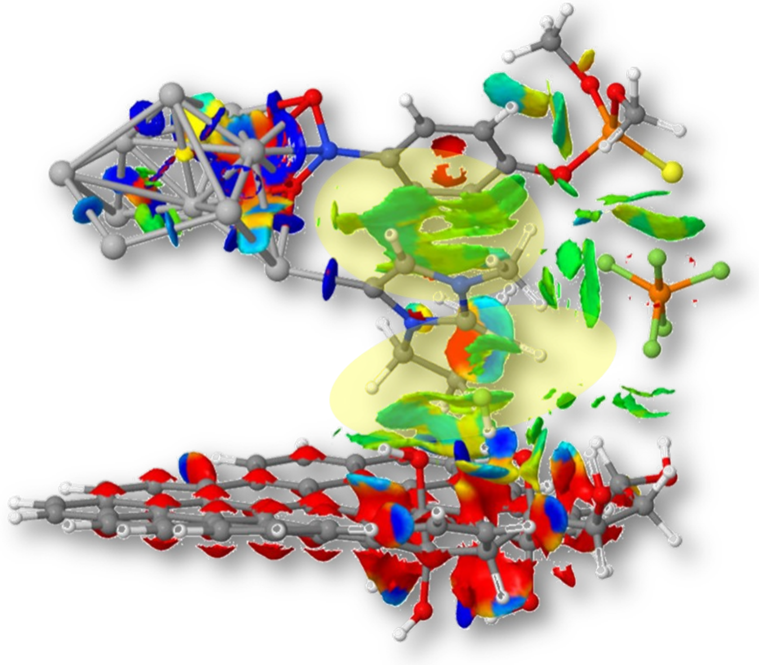

Figure S11. Non-covalent interaction domains in the (MP + Ag@GO/IL) system.
